# Discrepancy between Apolipoprotein E genotyping and proteotyping as a result of a nonsense mutation

**DOI:** 10.1002/alz.088844

**Published:** 2025-01-03

**Authors:** Ina Vandenbroucke, Tatsushi Yuri, Caroline Dobbels, Dagmara Minczakiewicz, Jo Kamada, Hisashi Nojima, Katsumi Aoyagi, Jasper Van Dongen, Fahri Kucukali, Kristel Sleegers, Rosina Degrieck

**Affiliations:** ^1^ Fujirebio Europe N.V., Ghent Belgium; ^2^ Fujirebio Inc., Tokyo Japan; ^3^ FUJIREBIO IINC., Hachioji, Tokyo Japan; ^4^ Complex Genetics of Alzheimer’s Disease group, VIB‐UAntwerp Center for Molecular Neurology, Antwerp Belgium; ^5^ Complex Genetics of Alzheimer’s Disease Group, VIB Center for Molecular Neurology, VIB, Antwerp Belgium

## Abstract

**Background:**

Apolipoprotein E (*APOE*) ε4 is a significant genetic risk factor for late‐onset Alzheimer’s Disease and appears to be closely related with brain amyloidosis. Current identification methods for *APOE ε4* carriers are mostly based on genotyping which cannot always predict the specific ApoE protein isoform. We present a case study of a sample with a discordant result for genotype compared to the protein isoform (proteotype) and we reflect on possible implications for future applications.

**Method:**

Proteotyping was performed on plasma using the Lumipulse® *G* Pan‐ApoE and Lumipulse® *G* ApoE4 <RUO> immunoassays on the LUMIPULSE *G* platform (Fujirebio, Japan). Isoelectric focusing electrophoresis (IEF) was performed using the Phenotyping ApoE IEF system (Joko Inc., Japan). Amplicon‐based sequencing of *APOE* on genomic DNA was performed by targeted long‐read sequencing on the Flongle platform using SQK‐LSK110 chemistry (Oxford Nanopore Technologies).

**Result:**

Previous comparison of genotyping (by real‐time PCR) with proteotyping on 230 paired blood /plasma samples revealed one discordant result (ε2/ε4 genotype versus negative E4 proteotype), achieving a 99.6% concordance rate. The proteotype of this discordant sample was confirmed to be E2 with absence of E4 using IEF. Long‐read gDNA sequencing of the discordant sample revealed a nonsense mutation (c.745G > T; p.E249X) in phase with the *APOE ε4* allele. This stop codon could either lead to a truncated protein, with a loss of function or altered function, or lead to degradation of the mRNA via the Nonsense Mediated Decay (NMD) pathway. Since the E4 isoform was not detected by the detecting antibody on the LUMIPULSE *G* platform, the most likely pathway is the degradation of the RNA by NMD, unless the E4 epitope was altered in the truncated protein.

**Conclusion:**

ApoE4 proteotyping offers advantages over genotyping by determining the effective presence of the protein in plasma, especially in case of mutations affecting the expression or function of the protein. Also, quantifying ApoE protein levels via immunoassays could yield insights into the relation of ApoE protein levels with pathology. Moreover, in case of potential future applications in a laboratory workflow, molecular testing could possibly be avoided, offering advantages in terms of turn‐around‐time and logistics.

